# International CCN society and intercellular signaling

**DOI:** 10.1186/1478-811X-5-1

**Published:** 2007-06-05

**Authors:** Bernard Perbal

**Affiliations:** 1 Laboratoire d'Oncologie Virale et Moleculaire, Case 7048, 2 place Jussieu, Universite Paris 7-D.Diderot, 75005, Paris, France

## Editorial

### International CCN society and intercellular signaling

When the first issue of Cell Communication and Signaling was launched in 2003, I had the opportunity to discuss the potential implications of CCN proteins in the control of cell-cell communication that is required for balanced tissue organisation, organ development, and species communication with their surrounding medium [1].

Since then, the CCN family of proteins has emerged as a central hub for extracellular matrix signaling and CCN proteins were found in different subcellular compartments where they interact with a variety of signaling ligands and receptors (see recent reviews in Perbal and Takigawa, 2005 [2]).

The fast pace at which the CCN field has developed in the past years, and the great variety of signaling pathways in which CCN proteins have been shown to play critical functions have both contributed to establish the International CCN Society (ICCNS) as an essential media for the exchange and diffusion of scientific information regarding inter cellular signaling.

The publication of the "Journal of cell Communication & Signaling [JCCS published by Springer]" as the official organ journal of ICCNS is major step along the establishment of the field.

For ethical reasons, the editorial board of CCS and myself had to present our resignation to BMC, effective september 2007.

We wish to inform that according to BMC policy manuscripts published in CCS will remain in the BMC database, and will remain freely accessible to readers.

The editorial board and myself wish to thank all authors for their contribution and members of the publishing team at BMC for their expertise and support during all these years.

Pr. Bernard Perbal

Editor in Chief
